# Experimental and computational studies of crystal violet removal from aqueous solution using sulfonated graphene oxide

**DOI:** 10.1038/s41598-024-54499-7

**Published:** 2024-03-14

**Authors:** Olayinka Oluwaseun Oluwasina, Adedeji Adebukola Adelodun, Olugbenga Oludayo Oluwasina, Helio A. Duarte, Sunday Joseph Olusegun

**Affiliations:** 1 Department of Marine Science and Technology, The Federal University of Technology, P.M.B. 704, Akure, 340110 Nigeria; 2 Department of Chemistry, University of Copenhagen, Universitet sparken 5, 2100 Copenhagen Ø, Denmark; 3 Department of Chemistry, The Federal University of Technology, P.M.B. 704, Akure, 340110 Nigeria; 4 Departamento de Química, Universidade Federal de Minas Gerais, Belo Horizonte, 31270-901 Brazil; 5 Department of Environmental Biotechnology, Faculty of Energy and Environmental Engineering, Silesian University of Technology, Gliwice, Poland; 6 Department of Chemistry, Michigan State University, 578 S. Shaw Lane, East Lansing, MI 48824-1322 USA

**Keywords:** Environmental sciences, Chemistry, Nanoscience and technology

## Abstract

Positively charged contaminants can be strongly attracted by sulfanilic acid-functionalized graphene oxide. Here, sulfonated graphene oxide (GO-SO_3_H) was synthesized and characterized for cationic crystal violet (CV) adsorption. We further studied the effect of pH, initial concentration, and temperature on CV uptake. The highest CV uptake occurred at pH 8. A kinetic study was also carried out by applying the pseudo-first-order and pseudo-second-order models. The pseudo-second-order’s adsorption capacity (qe) value was much closer to the experimental qe (qe_exp_:0.13, qe_cal_:0.12) than the pseudo-first-order model (qe_exp_:0.13, qe_cal_:0.05). The adsorption performance was accomplished rapidly since the adsorption equilibrium was closely obtained within 30 min. Furthermore, the adsorption capacity was significantly increased from 42.85 to 79.23%. The maximum adsorption capacities of GO-SO_3_H where 97.65, 202.5, and 196.2 mg·g^−1^ for CV removal at 298, 308, and 328 K, respectively. The Langmuir and Freundlich adsorption isotherms were applied to the experimental data. The data fit well into Langmuir and Freundlich except at 298 K, where only Langmuir isotherm was most suitable. Thermodynamic studies established that the adsorption was spontaneous and endothermic. The adsorption mechanism was revealed by combining experimental and computational methods. These findings suggest that GO-SO_3_H is a highly adsorbent for removing harmful cationic dye from aqueous media.

## Introduction

Colored water emerging from industrial activities, mainly from using dyes, is a major environmental threat^1^. Effluents from these industrial facilities are toxic and affect human health and aquatic life. The intense color of wastewater prevents sunlight from penetrating the water, resulting in the effluent having a higher chemical oxygen demand (COD)^2–4^. Crystal violet (CV), chemically known as *N*-[4-[bis[4-dimethyl-amino)-phenyl]-methylene]-2,5-cyclohexadien-1-ylidine]-*N* methylmethanaminium chloride, belongs to the category of triarylmethane-based dyes. It is characterized by its alkalinity and higher toxicity than anionic dyes^5^. CV has emerged as a persistent dye, recognized for its extended environmental presence and consequential toxic impacts. Within the textile industry, it serves as a purple dye, whereas in paper production, it is a vital component for navy blue-black hues for inks (in writing, painting, and printing)^6^. CV exerts detrimental influences on human health, posing severe eye irritation, skin disorders, respiratory issues, kidney failure, and even cancer^7^. Consequently, treating wastewater containing CV is imperative before discharge into the ecosystems.

Various approaches, such as advanced oxidation, membrane filtration, and photocatalytic degradation^8–12^, have eliminated this hazardous dye and ensured environmental safety. Nevertheless, diverse investigations have indicated that these strategies are not sufficiently reliable, simple, efficient, and affordable for removing dyes. Of the various removal techniques, adsorption is peculiarly excellent, owing to its remarkable attributes, including high efficiency, cost-effectiveness, and simplicity^4,13–15^. Biomass materials and nanomaterials have recently been widely investigated as potential adsorbents for dye removal with adsorption capacities of 16.094–280.2 mgꞏg^−1^^16–20^. Nonetheless, significant opportunities for improvement exist, particularly in addressing time and cost challenges in adsorbent synthesis, performance, and environmental pollution during dye removal^19^. Thus, devising innovative adsorbents with high adsorption capacity, cost-effectiveness, and environmental benignity is imperative to improving the availability of potable water.

Graphene, a carbon nanomaterial, exhibits low toxicity, a large specific surface area, and a single-layered aromatic conjugate plane structure, rendering it an excellent adsorbent for aromatics. Advancements in nanotechnology have facilitated the large-scale manufacturing of graphene. Recently, the interest in graphene and its derivatives as potential adsorbents for environmental remediation has surged^20–22^. However, graphene's limited dispersibility in water and tendency to aggregate due to its strong interplanar interactions remain challenging^23,24^. Graphene is often functionalized with surfactants or organic compounds to overcome these limitations. Functionalization has significantly enhanced graphene's adsorption capacity for various pollutants^25^.

The prevailing method for graphene synthesis commonly involves graphite oxidation, followed by exfoliation, resulting in graphene oxide (GO) formation. GO is a lamellar compound consisting of layers of carbon from graphene lattice that have been oxidized. Each GO layer is considered a multifunctional network, containing several oxygen functionalities and the carbon backbone. Due to its hydrophilicity, GO can easily be exfoliated and dispersed in water or organic solvents. The hydrophilicity of GO enables it to be evenly spread on the substrate in a single layer. The oxygen functional groups incorporated in the GO molecule produce reactive sites for chemical functionalization. GO requires additional functionalization to enhance its thermal stability, dispersibility, and compatibility with other substrates^26,27^. To improve its efficiency for dye removal, surface modification of graphene oxide (GO) is essential. Pristine GO may prove ineffective, especially regarding reusability, as numerous functional groups hinder the desorption of the dye from its surface. In this case, the sulfonated acid group is vital. Leveraging the solvation effect, this group can facilitate the dissociation of movable protons, thereby enhancing ionic conductivity. Compared to the carboxyl group, the sulfonated acid group demonstrates greater efficiency in proton dissociation and imparts a higher ionic exchange capacity to the substrate due to its stronger acidity^28,29^. Undoubtedly, GO functionalization with sulfonated acid groups can bolster ion exchange capacity and proton conductivity^29^. The negatively charged functional groups of GO-SO_3_H are expected to increase the adsorption capacity via the electrostatic and dispersion interactions with the positively charged CV dye. Therefore, we sulfonated GO via reaction with diazo salt of sulfanilic acid to obtain an enhanced adsorbent for the removal CV. The performance of the novel adsorbent was evaluated through a combination of experimental and computational methods, providing insights into the adsorption mechanism. The sulfonated graphene oxide (GO-SO_3_H) was modeled, permitting the investigation into six different adsorption sites and the importance of the carboxylic and sulfonic groups to the interaction of GO-SO_3_H with the crystal violet. On the chemical model, crystal violet adsorbed on various groups, such as sulfonic acid (–SO_3_H), carboxylic acid (–COOH), hydroxyl (–OH), μ-O, and μ-OH. Various techniques, including XRD, FTIR, SEM, and DFT calculations, were used to examine the microstructure-property relationship of the product. The research confirms the successful modification of sulfonated graphene oxide for the sorption of the cationic dye crystal violet.

## Experimental

### Materials

**Figure 1:**
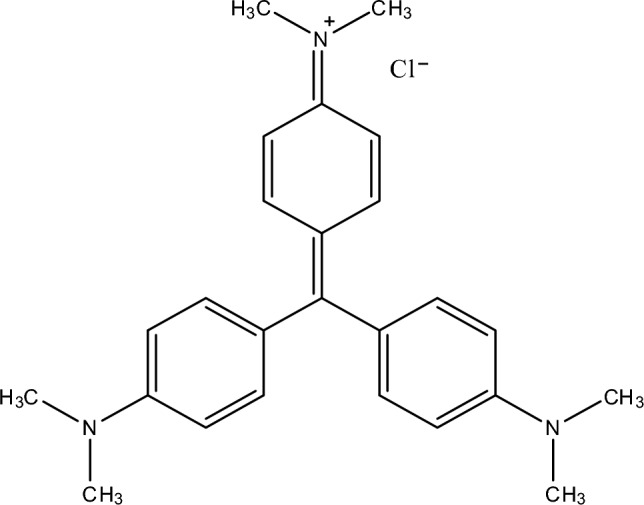
Chemical structure of crystal violet (molecular weight 407.98 g/mol, C_25_H_30_ClN_3_).

Graphite powder, sulfanilic acid, hydrochloric acid, sodium nitrate, sulphuric acid, potassium permanganate, sodium hydroxide, hydrogen peroxide, crystal violet, and sodium nitrite were purchased from Sigma-Aldrich and Minema chemicals (Johannesburg, South Africa). The molecular structure of CV is illustrated in Fig. 1. Various initial concentrations of the dye were prepared from a stock solution.

### Preparing GO and GO-SO_3_H

Graphene oxide (GO) was prepared using the method reported by Oluwasina et al.^30^. Briefly, GO (2 g), sodium nitrate (NaNO_3_) (1 g), and sulphuric acid (H_2_SO_4_) (46 mL) were mixed using a magnetic stirrer in an ice bath. Potassium permanganate (KMnO_4_) (6 g) was added gradually to keep the temperature below 5 °C. Then, it was removed from the ice bath, and the suspension was warmed to 35–40 °C for 90 min. The mixture was then heated to 98 °C for 20 min before adding 95 mL of distilled water. About 20 mL of 30% hydrogen peroxide (H_2_O_2_) was added to the mixture, which then turned yellow. 100 mL of distilled water was added to the mixture and agitated for 30 min. The product was centrifuged and washed with 20 mL of 10% hydrochloric acid (HCl) solution and distilled water. The final product was vacuum-dried for 48 h at 60 °C.

### Synthesis of sulfonated graphene oxide (GO-SO_3_H)

**Figure 2:**
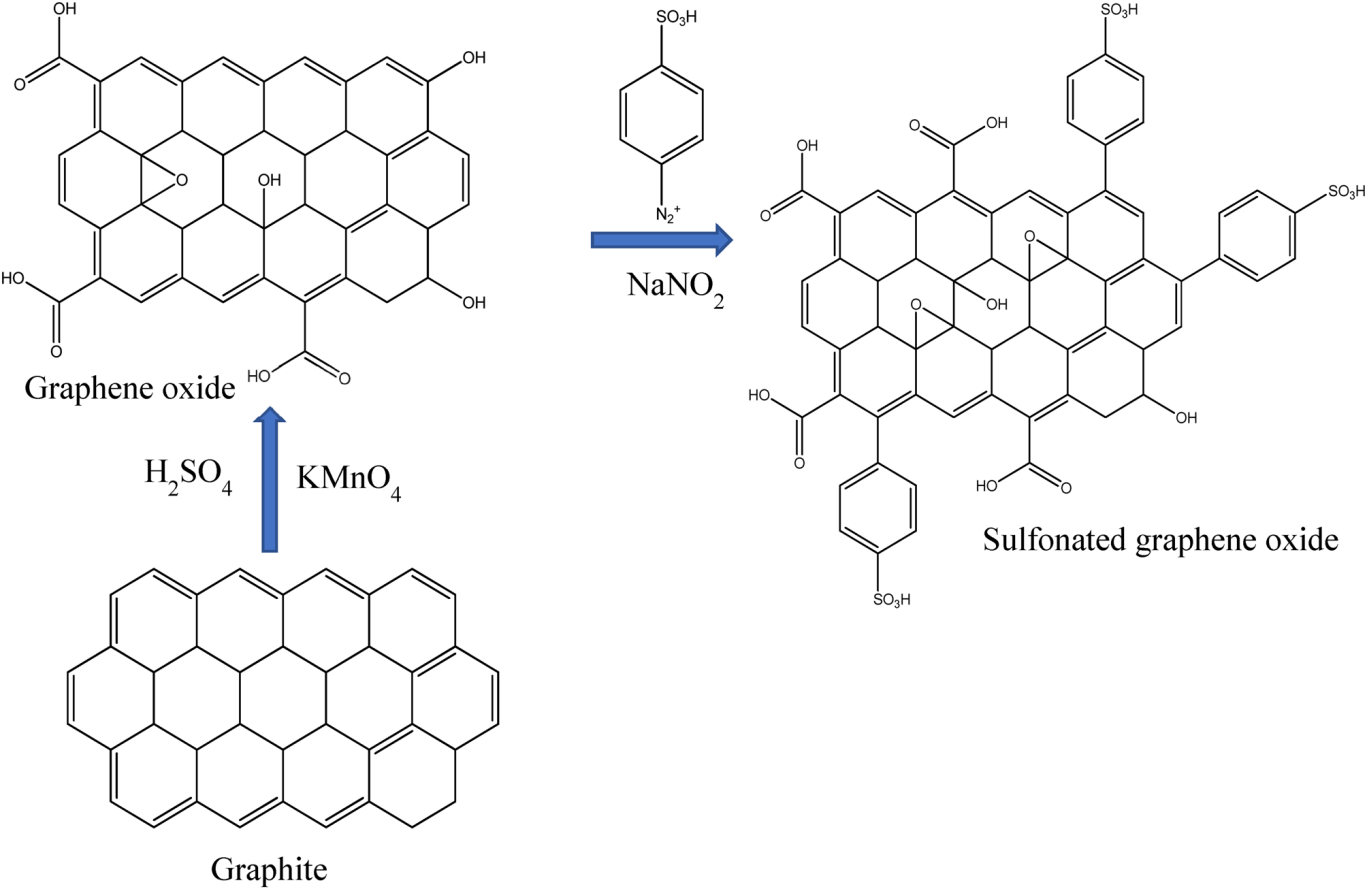
Schematic preparation of GO and GO-SO_3_H.

About 200 mg of GO was sonicated for 30 min in 200 mL of distilled water. A diazonium salt solution was prepared using 100 mg of 4-aminobenzenesulfonic acid (NH_2_C_6_H_4_SO_3_H), 40 mg of sodium nitrite (NaNO_2_), 1 mL of 1 N HCl and 10 mL of water. The mixture was slowly poured into the GO suspension and stirred at room temperature. The product was filtered, washed with distilled water, and vacuum-dried at 80 °C. The schemes for the GO and GO-SO_3_H syntheses are shown in Fig. 2.

### Characterization

Graphene oxide was sulfonated to enhance the surface affinity. The sample’s x-ray diffraction (XRD) pattern was examined (PANalytical Empyrean powder diffractometer, λ = 0.154 nm) using Cu Kα radiation with a Ni filter. The scan range and rate were 10°–100° and 2°/min, respectively. Fourier transform infrared (FTIR) spectrometry (QATR-S Shimadzu IRSpirit with single reflection ATR) was used to characterize the surface functional groups on the adsorbents. The surface morphology of GO and GO-SO_3_H was observed with scanning electron microscopy (SEM) (Tescan Vega LMH bearing an electron detector of 20.0 kV).

### Adsorption experiments

The CV adsorption was carried out using batch experiments. In brief, a predetermined amount of dried GO-SO_3_H (0.005–0.05 g) was added to 25 mL of 50–200 mg·L^−1^ of CV dye solutions. The adsorption medium pH was adjusted to pH 3–9, while the temperature was varied from 25 to 55 °C. Afterward, aliquots were taken at regular intervals (5 to 90 min) and filtered while the residual CV concentration was measured with an ultraviolet (UV) spectrophotometer at 592 nm. The following Eqs. (1, 2) were used to calculate the removal (%) and adsorption capacity (q):

1 $$\%=\frac{{C}_{o}-{C}_{e}}{{C}_{o}}\times 100$$

2 $${q}_{e}=\frac{({C}_{o}-{C}_{e})\times V}{w}$$

where *Co* and *Ce* signify CV concentration at the initial time *t*_*o*_ and definite time *t*, respectively, while *W* and *V* are the composite beads' weight and the CV dye solution volume, respectively.

### Computational methods

Density functional calculations were performed using the exchange/correlation function as regards Perdew, Berke, and Enzerhof (PBE)^31,32^ with the Def2-SVP basis sets^33^ as implemented in the ORCA version 5.0.3^34^. The RI approach was used with the auxiliary basis sets def2/J^35^. Empirical dispersion corrections from Grimme (D3)^36^ and the approximation resolution-of-identity (RI)^37–39^ were applied with the appropriate auxiliary basis sets to accelerate the calculations. The geometries were fully optimized with no constraints. The harmonic frequencies were calculated to estimate the Gibbs free energies and ensure that a minimum at the potential energy surface (PES) was found. The Hessian matrix was estimated numerically using a default increment of 0.005 Bohr. The solvation contribution to the Gibbs free energy has been estimated for the optimized gas-phase structures using the implicit solvation model called the SMD method proposed by Truhlar et al.^40^ with the solvent as water (${\varepsilon }_{{H}_{2}O}=78.355$). SMD is a universal solvation model that describes the solvent effects of charged or uncharged species based on the solute electron density without defining partial atomic charges.

**Figure 3:**
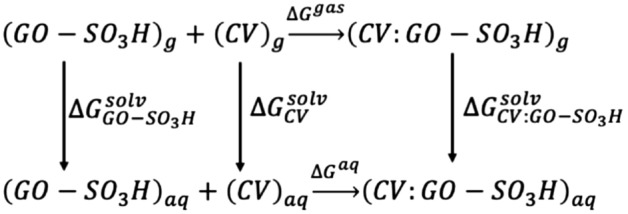
Thermodynamic cycle for calculating the Gibbs free energy of CV adsorption in aqueous solution.

The CV adsorption energy on GO-SO_3_H was estimated using the thermodynamic cycle (Fig. 3). The ΔG^gas^ for the adsorption at the gas phase was calculated using the ideal gas approximation and decomposed in the contributions (Eq. 3).

3 $$\Delta {G}^{gas}=\Delta {E}^{ele}+{\Delta H}^{therm}$$

The aqueous solution's adsorption Gibbs’ free energy in aqueous solution was estimated by adding the ΔΔG^solv^ according to Eq. (4).

4 $${\Delta G}^{aq}={\Delta G}^{gas}+{\Delta \Delta G}^{solv}$$

The GO-SO_3_H structure (Fig. 2) was the model for the sulfonated graphene oxide. Six different adsorption sites were proposed and calculated to estimate the importance of the carboxylic and sulfonic groups to the interaction of GO-SO_3_H with the CV. In the chemical model, CV adsorbed on various groups, such as –SO_3_H, –COOH, –OH, μ-O, and μ-OH (Fig. S1).

## Results and discussion

### Characterization of GO and GO-SO_3_H

#### FTIR

**Figure 4:**
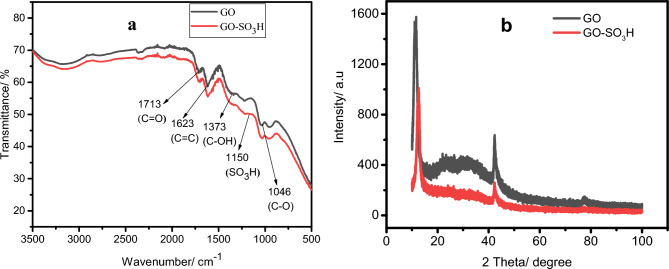
The FTIR (**a**) and XRD (**b**) of GO and GO-SO_3_H.

Figure 4a depicts the FTIR spectra of GO and GO-SO_3_H. Aside from the peak at 1623 cm^−1^, GO showed additional peaks associated with oxygen-containing groups. The peak at 1713, 1373, and 1046 cm^−1^ resulted from the stretching vibration of the carbonyl group (–C=O), bending vibration of the C–OH in H_2_O adsorbed in graphene oxide, and stretching vibration of the ether group (C–O)^41,42^, respectively. After further functionalization by sulfonation, sulfonated graphene oxide showed new additional peaks at around 1150 cm^−1^, corresponding to a sulfonated acid group^43^. Hence, graphite was successfully oxidized and sulfonated.

#### XRD

Further, XRD examined the crystalline structure of the prepared materials. The XRD pattern of GO and GO-SO_3_H is described in Fig. 4b. XRD analysis shows the discriminative peak of GO at 2θ = 10.95°, confirming the stretched interlayer spacing by incorporating some oxygen-containing groups, such as epoxy, carboxyl, and carbonyl^30^. Introducing the SO_3_H group onto GO during sulfonation shifted the characteristic peak of GO slightly from 2θ = 10.95° to 11.76° with a noticeable decline in the peak intensity as reported elsewhere^44^. This result confirms the sulfonation of GO to GO-SO_3_H.

#### SEM

**Figure 5:**
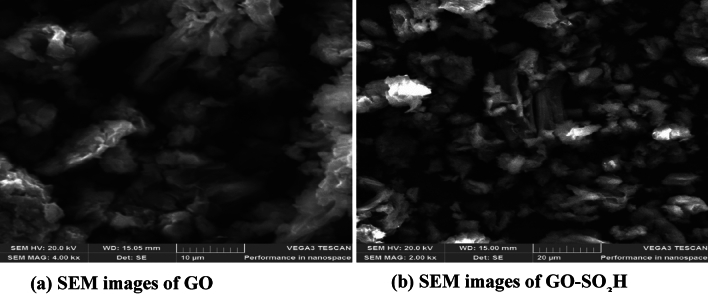
SEM images of GO and GO-SO_3_H.

Figure 5 displays the SEM images of GO and GO-SO_3_H. The resulting GO exhibits single-layer sheets with a significant thickness, a smooth surface, and a wrinkled edge. The GO-SO_3_H appears partially translucent and slightly folded, with isolated little bits of graphene on their surfaces^44^.

### Effect of pH

The adsorbent's surface characteristics, ionization or dissociation of the adsorbate molecule, and the interaction of the chelating adsorbent with the dyes were significantly impacted by the solution’s pH^45^. A report shows that the oxygen-containing groups on the graphene surface increase with increased adsorption of cationic pollutants^46^. The produced adsorbent has strongly ionizable sulfonic and carboxylic acid groups that can receive or donate protons depending on the surrounding pH. To better understand this process, GO-SO_3_H was used to adsorb CV. The CV solution's pH value largely determined the adsorption capacity.

**Figure 6:**
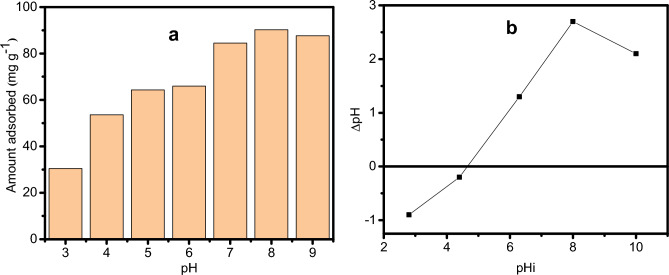
Effect of pH of CV adsorption (**a**) and the point of zero charge (pzc) of GO-SO_3_H (**b**).

Along with a pH increase from 3 to 8 (Fig. 6a), the adsorption capacity of GO-SO_3_H rose from 30.4 to 90.3 mg g^−1^. The point of zero charge (pHpzc) for GO-SO_3_H occurred at pH 4.6, and the adsorbent surface was negatively charged when the pH exceeded the pH_pzc_ (Fig. 6b). Hence, the amount of CV adsorbed increased when the surface charge of GO-SO_3_H tends toward negative, suggesting the adsorption was driven by electrostatic interactions between the dye molecule and the negatively charged surface of the adsorbents.

Expectedly, the electrostatic interaction between GO-SO_3_H and CV would be enhanced when pH increased because the sulfonic, carboxyl, and phenolic hydroxyl groups on GO-SO_3_H would deprotonate respectively, to form $R-{{\text{SO}}}_{3}^{2-}$, R-COO^−^, and R-O^−^ groups, leading to a negative surface. Thus, GO-SO_3_H adsorption capacity for CV increased quickly with pH. Also, H^+^ ions would compete with CV^+^ for exchangeable cations on the GO-SO_3_H in a very acidic solution, where the sulfonic groups would protonate to create sulfonic acid^47^. As a result, the electrostatic interactions between CV and GO-SO_3_H would be very weak, reducing CV adsorption onto GO-SO_3_H and leading to a low adsorption of CV. Conclusively, pH significantly impacted CV adsorption onto GO-SO_3_H.

Comparatively, the CV amount adsorbed at pH 8 was thrice that at pH 3. The adsorption efficiency was optimized at pH 8, reaching 72.22%. Hence, pH 8 was chosen as the optimum for further experiments.

At pH 3 and 4, where electrostatic repulsion is expected to occur due to the presence of two positive charges (adsorbent and adsorbate), 30 and 53 mg·g^−1^ of CV were adsorbed, respectively. This finding implies that the CV adsorption was driven by electrostatic attraction and other mechanisms. The insight into this was obtained from computational studies.

### Effect of dosage

**Figure 7:**
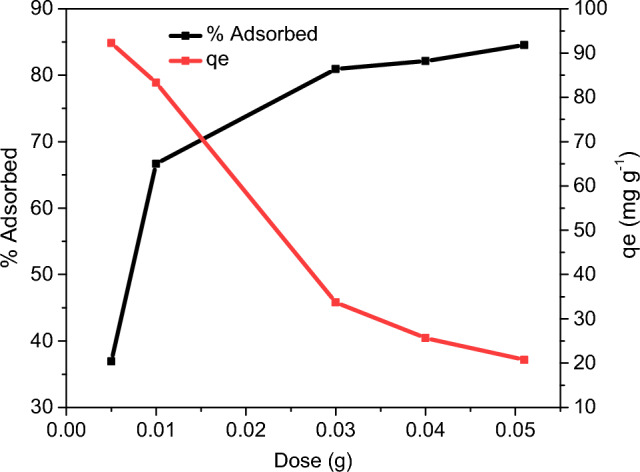
Effect of GO-SO_3_H dosage on CV adsorption.

The adsorption capacity was reduced dramatically by increasing the adsorbent dose from 0.005 to 0.05 g. This effect can be attributed to the increased adsorbent particles' propensity to aggregate, directly reducing the available surface area for the adsorption. As a result, more unoccupied active sites were present at a constant CV concentration, lowering the adsorption capacity value. On the other hand, as the adsorbent dosage was increased, CV removal rose slightly, reaching the maximum value at 0.04 g (Fig. 7). The increased sorbent’s surface area and availability of more adsorption sites are responsible for the significant initial increase.

### Adsorption kinetics

At the optimized pH and adsorbent dosage of 8 and 0.01 g, the GO-SO_3_H was added to the CV dye solution (25 mL, 50 mg‧L^−1^) for 5 to 90 min to evaluate the adsorption kinetics. We observed that the adsorption capacity at the initial stage (90.23 mg‧g^−1^ in the initial 20 min) indicated an excellent affinity between the sorbent and sorbate. Gradually, the exhaustion of adsorption sites caused the adsorption rate to equilibrate and plateau with time. The pseudo-first-order and pseudo-second-order models examined the adsorption rate and limiting factors.

**Figure 8:**
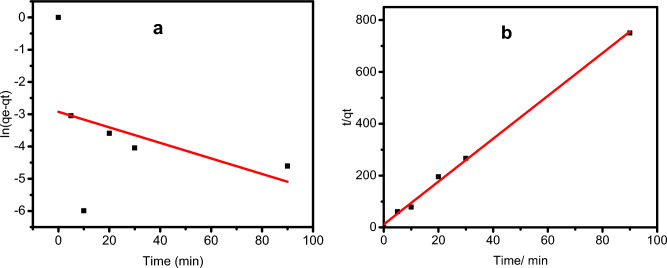
Pseudo-first-order (**a**) and pseudo-second-order (**b**) kinetic models for CV adsorption on GO-SO_3_H.

Table 1 displays the experimental and model values of the kinetic study. For the pseudo-first-order model, the qe, k1, and the correlation coefficient (R^2^) values were determined from the linear plot of ln(qe − qt) against t (Fig. 8a). The slope and intercept of the plot of t/qt against t (Fig. 8b) determined qe, k_2_, and R^2^ of the pseudo-second-order model. The pseudo-second-order model's R^2^ was > 0.99, while that of the pseudo-first-order indicates no fit. In addition, the pseudo-second-order’s qe value was much closer to the experimental qe than the pseudo-first-order model. These differences demonstrate that the pseudo-second-order kinetic model describes the adsorption more appropriately, i.e., the CV adsorption on GO-SO_3_H was not monolayer, and it was driven by chemical interactions at the interface.

**Table 1:** Parameters for kinetics study.

Model	Parameters	
Pseudo-first-order	qe(exp)	0.13
	qe(cal)	0.05
	K_1_	−0.00027
	R^2^	−0.051
	SSR	16.97
Pseudo-second-order	qe(cal)	0.12
	K_2_	5.887
	R^2^	0.9971
	SSR	855.96

### Effect of initial concentration

**Figure 9:**
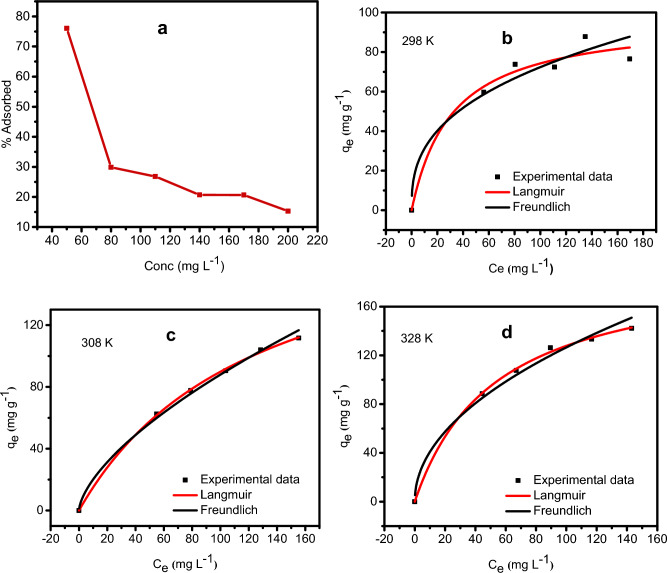
The effect of concentration on the adsorption of CV by GO-SO_3_H (**a**), adsorption isotherm at 298 K (**b**), 308 K (**c**), and 328 K (**d**).

Increased contaminant concentration renders its driving force stronger than the mass transfer resistance. As a result, more contaminating species migrated from the bulk solution onto the adsorbent^48^. We further investigated GO-SO_3_H (0.01 g) CV removal at 50–200 mg‧L^−1^ initial concentrations while keeping all other parameters constant. The CV adsorption efficiency decreased as the concentrations rose from 61.25 to 28.44% (Fig. 9a). The decrease could be attributed to the adsorption sites being constrained at excessively high contaminant concentrations. However, as the initial concentrations rose from 76.56 to 142.20 mg‧g^−1^, the adsorption capacity (qe) improved. This observation shows the adsorbent is excellent for removing cationic dye from wastewater.

### Adsorption isotherms

Adsorption isotherm studies detail the adsorption reaction pathways by describing how varied initial concentrations affect adsorption capacity and help discover the optimal equilibrium reactions. The Langmuir adsorption isotherm is based on the idea that molecule adsorbed occurs as a monolayer and posits that adsorption sites are similar and energetically equal:^49^, Eq. (5):

5 $${q}_{eq}=\frac{{q}_{m}b{C}_{eq}}{1+b{C}_{eq}}$$

The multilayer adsorption equilibrium on a heterogeneous surface is described empirically by the Freundlich model^50^, Eq. (6):

6 $${q}_{eq}={K}_{F}{C}_{eq}^{1/n}$$

where *q*_*eq*_ represents the adsorption capacity (mg‧g^−1^), *C*_*eq*_ denotes the adsorbate equilibrium concentration in solution (mg‧dm^3^), q_m_ represents the maximum monolayer capacity (mg‧g^−1^), *b* denotes the Langmuir isotherm constant (dm^3^‧mg^−1^), *K*_*F*_ denotes the Freundlich isotherm constant (mg‧g^−1^)(dm^3^‧mg^−1^), *n* denotes the adsorption intensity.

The adsorption isotherm modeling results in Table 2, Fig. 9b–d imply that the data fit well into Langmuir and Freundlich except at 298 K, where only Langmuir isotherm was most suitable. Using the Langmuir model, the modeled maximal adsorption capacities values (q_max_, cal) were relatively close to the experimental values (q_max_, exp). The comparison of the maximum adsorption capacity (q_max_) of different adsorbents for the adsorption of CV is presented in Table 3. However, the Freundlich parameter, n, shows the adsorption favorability. Usually, when the adsorption intensity n < 1, it shows that the adsorption is favorable across the whole concentration range examined; if n ˃ 1, it reveals that the adsorption intensity is favorable at high concentrations but lesser at lower concentrations. The experimentally determined n value for GO-SO_3_H was < 1 at 298, 308, and 328 K, indicating adsorption favorability over the investigated concentration range.

**Table 2:** Langmuir and Freundlich isotherms parameters for the adsorption of CV at 298, 308 and 328 K.

Parameters	298 K	308 K	328 K
Langmuir			
q_max_ (mg/g)	97.65	202.49	196.22
K_**L**_ (L/mg)	0.383	0.558	0.711
R^2^	0.964	0.999	0.998
Freundlich			
K_F_	24.62	6.685	20.702
n_F_	0.237	0.561	0.392
R^2^	0.445	0.993	0.963

**Table 3:** Comparison of the maximum adsorption capacity (q_max_) of GO-SO_3_H with various adsorbents used for CV adsorption.

Adsorbents	q_max_ (mg/g)	References
Iron oxide (Fe_3_O_4_) magnetic nanoparticles	66.01	^51^
Tectona grandis sawdust	131.58	^52^
Zirconium oxide/activated carbon (Zr_3_O/AC) composite	204.12	^53^
Magnetite alginate	37.5	^54^
Poly(acrylamide)-kaolin composite	23.8	^55^
Biomaterial-based flower-MnO2@ carbon microspheres	16.094	^16^
Graphene oxide-based sponges	280.2	^20^
Chitin nanowhiskers	59.52	^56^
GO-SO_3_H	202.49	This work

### The influence of temperature and adsorption thermodynamics

**Figure 10:**
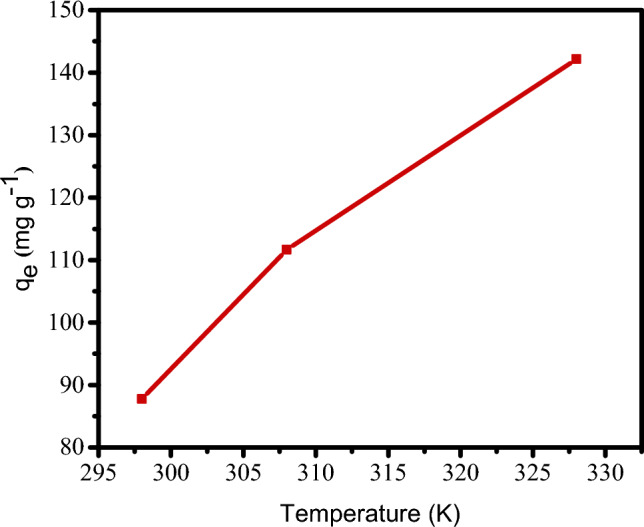
Influence of temperature on CV adsorption on GO-SO_3_H.

Figure 10 illustrates how increased temperature affects GO-SO_3_H’s adsorption of CV. The CV removal increased with temperature (Fig. 10), exhibiting an endothermic reaction characteristic of adsorption driven by chemical reactions. As the temperature increased, the CV molecules gained more kinetic energy, hastening their movement toward the adsorption sites, resulting in chemisorption^57^.

These findings show a significant interaction between CV and GO-SO_3_H, such that the adsorbed molecules would not desorb by increased temperature until the activation energy was surpassed. It also demonstrated that the adsorption equilibrium was altered when the temperature rose, favoring CV adsorption. El Kady et al.^58^ asserted that temperature is essential to adsorption because it determines the mobility of the adsorbate molecules and generates new binding sites for their adsorption.

We gain further insights into the adsorption thermodynamics by applying Eqs. (7–9) to the experimental data. The Langmuir adsorption equilibrium (K_L_) constant was calculated using Eq. (7), after which the three fundamental thermodynamic parameters (i.e., Gibbs free energy change (*ΔG°*), enthalpy change (*ΔH°*), and entropy change (*ΔS°*)) were calculated using Van't Hoff and Gibbs–Helmholtz equations (Eqs. (8) and (9)).

7 $${K}_{L}=\frac{{q}_{e}}{{C}_{e}}$$

8 $$ln{K}_{L}=\frac{\Delta {\text{S}}^\circ }{R}-\frac{\Delta {\text{H}}^\circ }{RT}$$

9 $$\Delta {\text{G}}^\circ =\Delta {\text{H}}^\circ -{\text{T}}\Delta {\text{S}}^\circ$$

where K_L_ is the adsorption equilibrium constant, q_e_ is the equilibrium adsorption capacity, C_e_ is the equilibrium concentration.

The adsorption thermodynamics was investigated at 298, 308, and 328 K. The negative *ΔG°* values govern the viability and spontaneity of the sorption. The negative *ΔG°* values (−13.73 to −15.11 kJ‧mol^−1^ in Table 4) indicated that the adsorption of CV onto GO-SO_3_H was spontaneous and thermodynamically favorable. As the temperature rose, the negative values of *ΔG°* increased, indicating improved adsorption. The positive ΔH° value (15.97 kJ‧mol^−1^) suggested the endothermic nature of the adsorption, in which an increase in temperature favors the adsorption uptake of CV. According to the values of ΔH° for CV onto GO-SO_3_H, the sorption process was a chemisorption. The positive ΔS° of the adsorbent implies that the adsorption was entropy-driven.

**Table 4:** Thermodynamics parameters of the adsorption of CV onto GO-SO_3_H.

Adsorbate	T (K)	ΔG° (kJ‧mol^−1^)	ΔH° (kJ‧mol^−1^)	ΔS° (J‧K^−1^‧mol^−1^)
CV	298	−13.734		
	308	−14.196	15.971	46.142
	328	−15.118		

### DFT calculations

**Figure 11:**
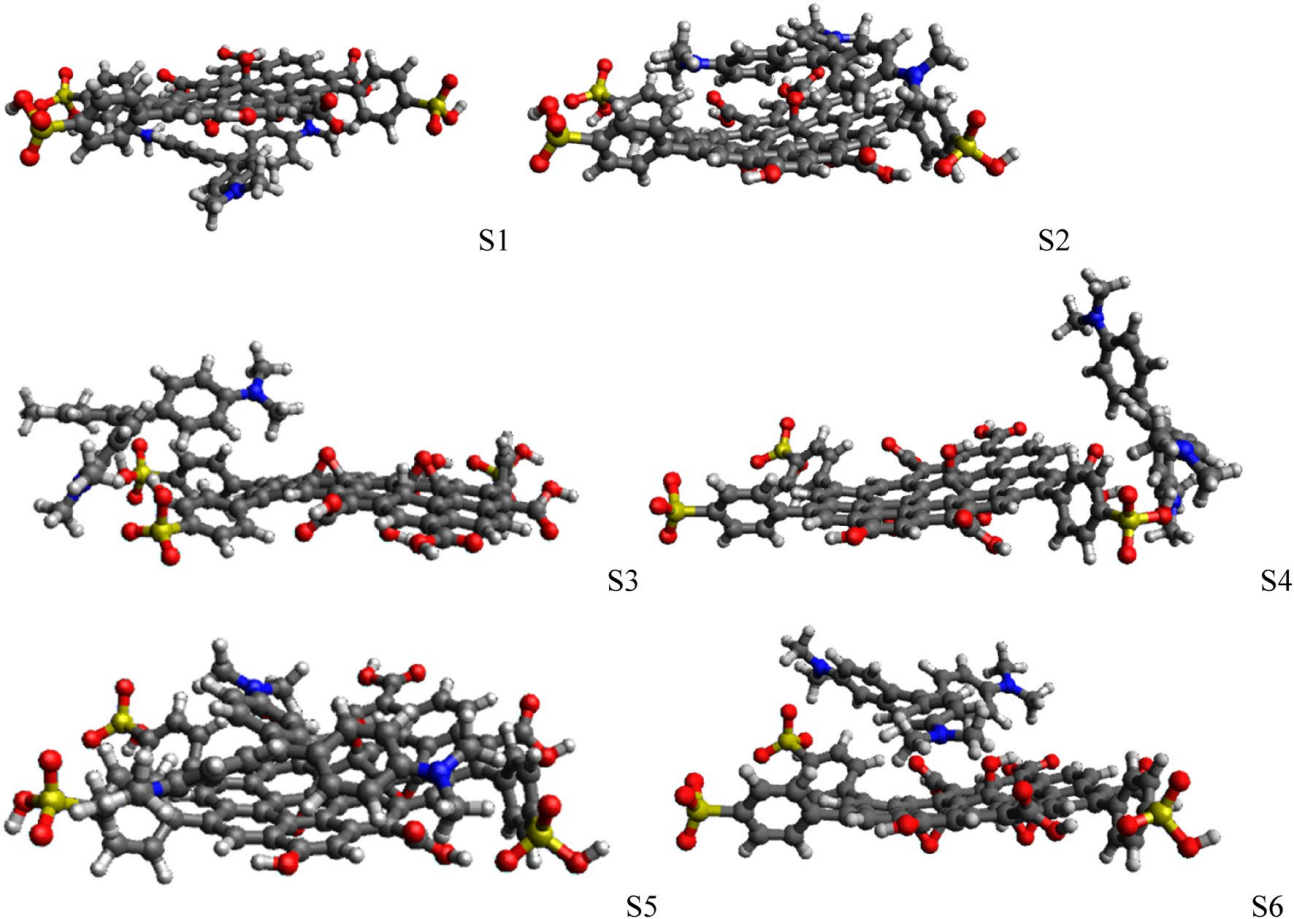
Optimized structures for six different adsorption sites for charge + 1 species. Black is carbon, red is oxygen, blue is nitrogen, yellow is sulfur, and white is hydrogen. The figure was created using Avogadro 1.2: (https://sourceforge.net/projects/avogadro/).

The adsorption energy was estimated for six adsorption sites on the GO-SO_3_H model (Fig. 11). Table 5 shows the two most favorable sites. We investigated the adsorption using the GO-SO_3_H deprotonated at the carboxylic and sulphonic groups, causing the CV:GO-SO_3_H structures to exhibit + 1, 0, and −1 charges. Sites 2 and 5 were the most favorable (Fig. 11). We also observed that the deprotonation increased the negative charge on the GO-SO_3_H. However, it does not imply that the adsorption energy increased. We opine that the solvation was fundamental to the adsorption. Apparently, the electrostatic interaction was predominant with the increased system charge. It was a tradeoff between the solvation energy of the reactants and their interaction. The CV presented a + 1 charge, while GO-SO_3_H presented 0, −1, and −2 charges according to the degree of deprotonation. Forming CV:GO-SO_3_H lowered the charge and, consequently, the solvation energy of the process (${\Delta \Delta G}^{solv}$) decreased while the electrostatic interaction of the system increased $({\Delta G}^{gas})$. Note that ${\Delta \Delta G}^{solv}$ is estimated based on a continuum model of the solvent. The explicit solvent effects, such as the hydrogen bonding of the water solvent and the carboxylic or sulfonic groups, were neglected. The water molecules are released upon adsorption to permit the CV:GO-SO_3_H interaction, increasing the system's entropy. Our results show that the CV:GO-SO_3_H interaction was predominantly due to dispersion interaction, recovered by the Grimme D3 approximation added in the calculations.

**Table 5:** DFT adsorption free energies (in kJ‧mol^−1^) for CV adsorbed in different sites on the GO-SO_3_H model in aqueous solution. 1 (1 calculations performed at the PBE-D3/def2-SVP/SMD level of theory).

Deprotonated groups	Charge	Sites	ΔE_gas_	ΔH_Therm_	ΔΔG_solv_	ΔG^aq^_ads_
–COOH –SO_3_H	Charge + 1	S2	−207.45	89.24	43.26	−74.94
		S5	−213.10	93.85	47.78	−71.47
–COO^−^ –SO_3_H	Charge 0	S2	−388.96	94.63	239.11	−55.22
		S5	−351.58	91.48	186.40	−73.70
–COOH –SO_3_^−^	Charge 0	S2	−390.26	91.04	221.90	−77.32
		S5	−386.12	93.53	220.66	−71.94
–COO^−^ –SO_3_^−^	Charge −1	S2	−558.46	94.35	408.48	−55.63
		S5	−532.36	94.69	368.81	−68.86

To verify this hypothesis, we have performed the same calculations for the S5, but without D3 contribution. The $\Delta {G}_{g}^{ads}(=\Delta {E}_{g}^{ele}+\Delta {H}_{0}^{therm})$ was estimated to be exergonic with only −9.83 kJ/mol, compared to −119.3 kJ/mol using D3 approach. Adding the ${\Delta \Delta G}^{solv}$ of 47.78 kJ/mol, the adsorption became endoergonic with 38 kJ/mol. The deprotonation of the groups increased the delocalization of charges. Hence, the dispersion interaction must be added to describe the adsorption process correctly.

The effect of varied temperatures was also evaluated. Table S2 lists the adsorption free energy for the various temperatures. As expected, the entropy increased with temperature, hence lowering the adsorption energy for the site S5 from −71.9 kJ/mol at 298 K to −69.6 and −63.8 kJ/mol at 308 and 328 K, respectively. One possible explanation is that the adsorbent presents acid groups that can be deprotonated depending on pH variation. The pKa decreased upon increasing temperature, augmenting the charge on the system and favoring CV interaction with GO-SO_3_H. A 0.2 decrease in the pKa is expected to accompany the temperature rise to 308 K.

## Conclusion

We successfully synthesized sulfonated graphene oxide (GO-SO_3_H) and applied it for crystal violet (CV) adsorption from an aqueous solution. The results showed that GO-SO_3_H can effectively and adequately remove CV. Based on the effect of solution pH, CV adsorption was favored by increased pH, optimized at the pH of 8. Further, pseudo-second-order best describes the adsorption kinetic. The data fit well into Langmuir and Freundlich except at 298 K, where only Langmuir isotherm was most suitable. The increase in temperature of the aqueous solutions favored the spontaneity of CV removal. The achieved maximum adsorption capacity (202 mg‧g^−1^) was favored by electrostatic and dispersion interactions, confirmed by experimental and computational studies. Consequently, sulfonated graphene oxide is an excellent adsorbent for crystal violet.

### Supplementary Information

Supplementary Information.

## Data Availability

The data presented in this study are available on request from the corresponding author.
