# Supplementary material for: Experimental and computational studies of crystal violet removal from aqueous solution using sulfonated graphene oxide
Source: Sci Rep. 2024 Mar 14;14:6207. doi: 10.1038/s41598-024-54499-7 (PMC10940666; doi:10.1038/s41598-024-54499-7)
Supplement: Supplementary file 1 — Supplementary Information. [file 41598_2024_54499_MOESM1_ESM.pdf]

Table S1- DFT adsorption free energies (in kJ mol^{-1}) for CV adsorbed in different sites on the GO-SO_3H model in aqueous solution.1

Deprotonated groups	Charge	Sites	ΔE_{gas}	Δ_{Therm}	ΔG_{solv}	ΔG_{ads}
-COOH		S1	-148.76	77.46	13.15	-58.15
-SO_3H		S2	-207.45	89.24	43.26	-74.94
		S3	-140.00	90.94	37.96	-11.10
	Charge +1	S4	-109.64	79.54	16.09	-14.01
		S5	-213.10	93.85	47.78	-71.47
		S6	-182.37	96.08	40.70	-45.59
-COO^-		S1	-350.08	82.67	206.12	-61.29
-SO_3H		S2	-388.96	94.63	239.11	-55.22
		S3	-269.96	87.15	165.73	-17.08
	Charge 0	S4	-246.36	75.85	150.23	-20.28
		S5	-351.58	91.48	186.40	-73.70
		S6	-360.10	93.76	216.37	-49.98
-COOH		S1	-335.15	78.32	217.08	-39.75
-SO_3^-		S2	-390.26	91.04	221.90	-77.32
		S3	-239.85	83.85	132.79	-23.21
	Charge 0	S4	-256.67	76.56	200.52	20.41
		S5	-386.12	93.53	220.66	-71.94
		S6	-325.41	84.55	206.13	-34.72
-COO^-		S1	-496.06	79.11	357.93	-59.01
-SO_3^-		S2	-558.46	94.35	408.48	-55.63
		S3	-387.31	80.28	282.92	-24.12
	Charge -1	S4	-546.84	88.32	399.58	-58.95
		S5	-532.36	94.69	368.81	-68.86
		S6	-396.69	85.30	293.45	-17.94

1 Calculations performed at the PBE/def2-SVP/SMD level of theory.

Table S2 – Estimated thermodynamic values in kJ mol^{-1} for the adsorption site S5.

Deprotonated groups	Charge	ΔG_g^{ads}	ΔH_g^{ads}	ΔS_g^{ads}	$\Delta\Delta G_{Solv}^{1}$	ΔG_{aq}^{ads}
		298 K				
-COOH -SO_3H	+1	-120.3	-206.3	-86.0	47.8	-72.5
-COO^- -SO_3H	0	-260.3	-345.4	-85.1	186.4	-73.9
-COOH -SO_3^-	0	-439.2	-524.1	-84.9	368.8	-70.3
-COO^- -SO_3^-	-1	-293.3	-376.4	-83.1	220.7	-72.6
		308 K				
-COOH -SO_3H	+1	-117.4	-206.2	-88.8	47.8	-69.6
-COO^- -SO_3H	0	-257.5	-345.3	-87.8	186.4	-71.1
-COOH -SO_3^-	0	-436.3	-524.0	-87.7	368.8	-67.5
-COO^- -SO_3^-	-1	-290.5	-376.3	-85.8	220.7	-69.8
		328 K				
-COOH -SO_3H	+1	-111.6	-206.0	-94.4	47.8	-63.8
-COO^- -SO_3H	0	-251.7	-345.1	-93.4	186.4	-65.3
-COOH -SO_3^-	0	-430.6	-523.8	-93.2	368.8	-61.8
-COO^- -SO_3^-	-1	-284.8	-376.0	-91.2	220.7	-64.2

[1] The variation of the solvation free energies calculated using the SMD was estimated only at 298K.

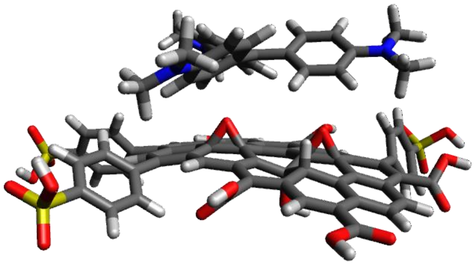

S1

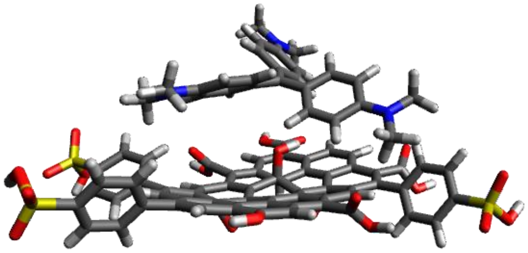

S2

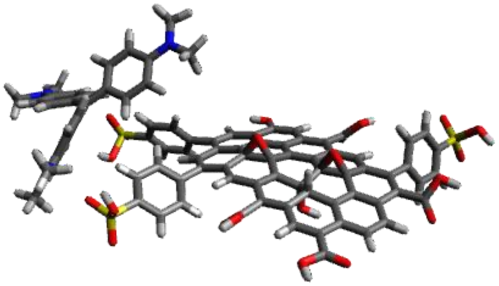

S3

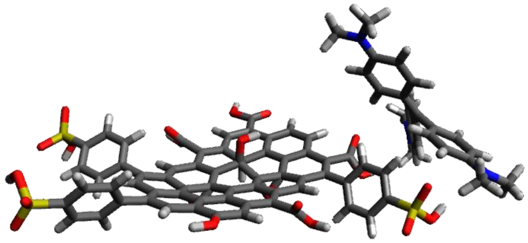

S4

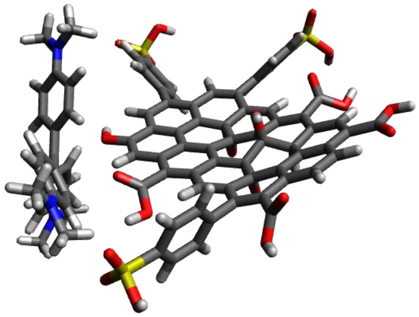

S6

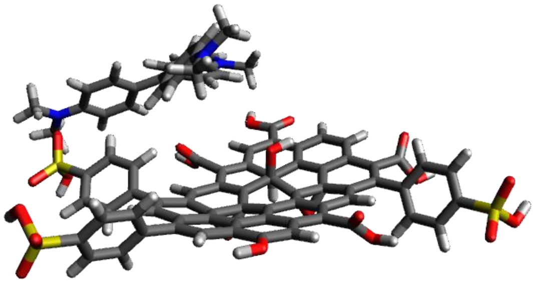

S5

Figure S1: Initial structures for geometry optimization at the six different adsorption sites. The figure was created using Avogadro 1.2: (https://sourceforge.net/projects/avogadro/)
